# Effectiveness of Conversational Agents on Patient-Reported Outcomes in Chronic Pain Management: A Systematic Review and Meta-Analysis

**DOI:** 10.3390/healthcare14101360

**Published:** 2026-05-15

**Authors:** Jesús Zamora-Tortosa, Alejandro Heredia-Ciuró, Carmen Cruz Herrera, Rafael Jiménez López, Jiawei Guo Liang, Marie Carmen Valenza, Eva Lantarón-Caeiro

**Affiliations:** 1 Department of Physiotherapy, Faculty of Health Sciences, University of Granada, Av. de la Ilustración 60, 18016 Granada, Spain; jzamoratortosa@correo.ugr.es (J.Z.-T.); ccruzherrera@correo.ugr.es (C.C.H.); rafajlopez@correo.ugr.es (R.J.L.); andreguo@correo.ugr.es (J.G.L.); cvalenza@ugr.es (M.C.V.); 2 Faculty of Physiotherapy, University of Vigo, Campus A Xunqueira, 36005 Galicia, Spain; evalantaron@uvigo.gal

**Keywords:** chronic pain, conversational agents, chatbots, patient-reported outcome measures, telehealth, systematic review, meta-analysis

## Abstract

**Highlights:**

**What are the main findings?**

- CA-based interventions improve patient-reported outcomes in adults with chronic pain, with the most consistent effects observed for pain intensity.
- CA-based intervention may present beneficial effects across psychological and functional outcomes.

**What are the implications of the main findings?**

- Conversational agents may offer a scalable approach to support symptom monitoring, self-management, and behavioral guidance in chronic pain care.
- Interventions that incorporate frequent, structured, and interactive delivery formats may be particularly suitable for maximizing patient engagement and clinical benefit.

**Abstract:**

**Background:** Chronic pain remains a primary driver of global disability and impaired quality of life. While digital conversational agents (CAs) have emerged as scalable tools for symptom monitoring and self-management via patient-reported outcome measures, their clinical efficacy remains poorly synthesized. This systematic review and meta-analysis aimed to evaluate the impact of CA-based interventions on PROMs in adults with chronic pain. **Methods:** A systematic review and meta-analysis was conducted following PRISMA 2020 guidelines. PubMed, Scopus, and Web of Science were searched from inception to 22 October 2025. Eligible studies were RCTs including adults with chronic pain and evaluating fully automated CA interventions, such as digital coaching or messaging programs. PROMs related to pain, well-being, disability, and work-related outcomes were extracted. Continuous outcomes were synthesized using standardized mean differences (SMDs) with 95% confidence intervals (CIs). **Results:** Five RCTs involving 572 participants were included. Interventions were self-guided, digitally delivered, and lasted 4 to 12 weeks. The overall pooled analysis suggested a potential benefit of CA-based interventions on PROMs (SMD = −0.43; 95% CI −0.55 to −0.31; *p* < 0.00001), although heterogeneity and risk of bias across studies warrant cautious interpretation. Improvements were observed particularly in pain intensity, although evidence for other outcomes was less consistent, with some studies reporting benefits in quality of life, fear of movement, and well-being. **Conclusions:** CA-based interventions may have potential as adjuncts in chronic pain management; however, the current evidence is limited and should be interpreted with caution due to heterogeneity and risk of bias across studies. These tools may represent a scalable solution for supporting remote symptom monitoring and self-management within digital health frameworks, although further high-quality evidence is required.

## 1. Introduction

Chronic pain is commonly defined as pain that persists or recurs for more than 3 months [1]. It is widely recognised as a major global health problem because of its considerable contribution to disability and high prevalence [2]. In Europe, a recent meta-analysis estimated a prevalence of 21.5%, and approximately 20.5% has been reported in the United States [3,4]. Given the substantial proportion of people affected, chronic pain is associated with impaired functioning, frequent comorbidities such as depression and anxiety—which affect up to 50% of this population—and reduced productivity [2]. Beyond the personal impact, it results in a massive economic burden; recent estimates suggest that the total cost of chronic pain (including healthcare utilization and productivity loss) accounts for 2% to 3% of the total GDP in European countries, representing an annual cost of approximately €450 billion across the EU [5].

In response to this multidimensional burden, clinical guidelines and reference literature recommend a personalised and multimodal management approach that combines pharmacological and non-pharmacological strategies [1,5]. However, routine practice can limit access to individualised multidisciplinary care and result in waiting times of several months [6]. These delays may contribute to persistent pain, greater pain-related disability, and increasingly widespread pain over time among people with chronic pain [7,8].

To support personalized and individualized therapeutic management, healthcare providers are continuously in search of cost-effective patient-centered care strategies [9]. A core element of chronic patient-centered care is the assessment of patient-reported outcomes (PROs), defined as any aspect of a patient’s health status reported by the patient [10,11]. Patient-reported outcome measures (PROMs) are particularly relevant in chronic pain because they quantify the patient-perceived impact of pain on daily life and wellbeing and enable structured tracking of these domains over time [12,13].

The search for cost-effective patient-centered care strategies has contributed to growing interest in digital health as a potential complement to face-to-face care [14,15]. Recent reviews suggest that digital approaches may improve patients’ understanding of their condition, motivation, and perceived continuity of support [16,17]. From a theoretical perspective, digital health interventions can be situated within behavioural change frameworks such as Social Cognitive Theory, Self-Determination Theory, and the COM-B model, which emphasize the role of capability, opportunity, and motivation in sustaining health behaviours [18]. However, the effectiveness of digital approaches may depend not only on the therapeutic content but also on intervention design and implementation features. Engagement, adherence, and sustained self-management are therefore key mechanisms through which digital interventions may exert their effects, as described in digital health engagement frameworks and persuasive system design models [19]. In this context, conversational agents (CAs) have gained increasing attention as interactive telehealth technologies that can provide accessible and tailored support for chronic pain. Within this theoretical context, CAs can be understood as conversational systems designed to operationalize behavioural support principles through natural language interaction, enabling continuous feedback, reinforcement, and self-monitoring [20]. In addition to providing ongoing support, CAs represent promising interventions for chronic pain management [20,21] and can facilitate the frequent, low-burden collection of PROMs, thereby supporting patient-centered monitoring in real-world settings.

Despite increasing interest in CAs as telehealth technologies for chronic pain management, to our knowledge no systematic review has comprehensively synthesized randomized evidence in adults with chronic pain. Furthermore, key intervention characteristics and methodological features associated with outcomes have not been systematically characterized [22]. We therefore conducted this systematic review to synthesize the available evidence and provide an integrated overview of the current state of knowledge. The aim was to evaluate the effectiveness of CAs in adults with chronic pain, based exclusively on randomized controlled trials, and to characterize intervention characteristics and methodological variables associated with outcomes to inform clinical practice and guide the design of future interventions.

## 2. Materials and Methods

### 2.1. Study Registration

To address the aim of this review, the Preferred Reporting Items for Systematic Reviews and Meta-Analyses (PRISMA) 2020 statement was followed [23]. A protocol for this systematic review and meta-analysis was prospectively registered in PROSPERO (International Prospective Register of Systematic Reviews) under registration number: CRD420251245189 (4 December 2025).

### 2.2. Search Strategy

We carried out a comprehensive literature search across the following databases: PubMed, Scopus, and Web of Science, from inception to December 2025. The final search was performed on 22 December 2025. The search strategy was based on MeSH terms and keywords related to conversational agents and AI-based interactive systems, including terms such as “chatbot”, “conversational agent”, “conversational AI”, “virtual agent”, “avatar”, “digital assistant”, and “natural language interface”, combined with terms related to chronic pain and persistent musculoskeletal pain conditions, such as “chronic pain”, “non-cancer pain”, “musculoskeletal pain”, “osteoarthritis”, “arthritis”, “myalgia”, and “myofascial pain syndrome”.

To ensure that the search strategy was as complete as possible, the terms were refined and adapted for each database. No database-specific methodological filters were applied beyond the predefined eligibility criteria. The full search strategy for all databases, including the specific Boolean terms and filters applied, is available as Supplementary Materials (Figure S1).

### 2.3. Study Selection

Eligibility criteria were defined according to the PICOS framework [24]. Studies were considered eligible if they met the following criteria: P (Participants): adults with chronic pain conditions persisting for more than 3 months; I (Intervention): fully automated conversational agents, including chatbot-based interventions or structured digital coaching/messaging programs designed to provide education, self-management support, symptom monitoring, or related behavioral guidance; C (Comparison): interventions without automated conversational agents or no intervention; O (Outcomes): patient-reported outcomes (PROMs), including pain intensity, pain-related disability, mental well-being, self-efficacy, and other health-related outcomes; S (Study design): randomized controlled trials.

No language restrictions were applied in order to minimize selection bias. To ensure comprehensive coverage of the literature, we additionally searched trial registries, preprints, and grey literature sources, and screened the reference lists of included studies and relevant systematic reviews.

Studies that did not meet the inclusion criteria or were not randomized controlled trials were excluded. Duplicate records were removed prior to screening. After duplicate removal, two reviewers (J.Z.T. and J.G.L.) independently screened titles and abstracts and then assessed the full texts of potentially eligible studies. Any disagreements were resolved through discussion and, when necessary, consultation with a third reviewer (M.C.V.).

### 2.4. Data Extraction

The following data were extracted: first author, year of publication, country, study design, sample size and participant characteristics, clinical condition, intervention characteristics, comparator details, intervention duration, follow-up period, outcome measures, and main findings.

When data were unclear or incomplete, the corresponding authors were contacted by e-mail. Two reviewers extracted the data independently, and any disagreements were resolved through discussion with a third reviewer.

### 2.5. Qualitative Synthesis and Quality Assessment

The methodological quality of the included studies was assessed using the Downs and Black checklist [25], which covers reporting quality, external validity, internal validity, and study power. Each study included was independently assessed by two reviewers, and disagreements were resolved through discussion or consultation with a third reviewer. Scores were summarized as total study scores, with additional consideration of domain-specific performance.

In addition, the completeness of reporting of the digital interventions and trial procedures was assessed using the CONSORT-EHEALTH checklist [26]. This was used to examine how well the included trials reported key eHealth-specific methodological and intervention details. The results were summarized by assigning each study a score based on the number of applicable CONSORT-EHEALTH items addressed.

Risk of bias was independently assessed by two reviewers using the Cochrane Risk of Bias 2 (RoB 2) tool for randomized controlled trials [27]. This tool evaluates five domains: bias arising from the randomization process, deviations from intended interventions, missing outcome data, measurement of the outcome, and selection of the reported result. Studies were judged as having low risk of bias, some concerns, or high risk of bias.

The certainty of the evidence for the primary outcomes (pain intensity, anxiety, depression, and stress) was assessed using the GRADE (Grading of Recommendations Assessment, Development and Evaluation) approach. Evidence was classified into four levels of certainty: high, moderate, low, or very low. The assessment considered five specific domains: risk of bias, inconsistency (heterogeneity), indirectness, imprecision (confidence intervals), and publication bias. Discrepancies in the grading process were resolved through consensus among the research team.

### 2.6. Meta-Analysis

Meta-analyses were conducted using Review Manager (RevMan) version 5.4, developed by the Cochrane Collaboration [28]. For each outcome, quantitative data were extracted for both experimental and control groups, including the number of participants analyzed, post-intervention means, and standard deviations. Standardized mean differences (SMDs) were applied when different scales were used to assess the same construct. All effect estimates are presented with their respective 95% confidence intervals. Given the clinical heterogeneity among the included outcomes, the overall pooled effect estimate was considered exploratory and intended to provide a broad summary of intervention effects across presented domains.

Before pooling, outcome directions were checked to ensure a consistent interpretation of effect estimates across studies. Statistical heterogeneity was assessed using the I^2^ statistic. A fixed-effect model was applied when I^2^ was below 50%. A random-effects model was used for the quantitative synthesis given the expected clinical and methodological heterogeneity across the included studies.

When quantitative synthesis was not considered appropriate because of differences in interventions, outcome measures, or reporting formats, findings were compared narratively within the qualitative synthesis. When necessary, missing standard deviations were calculated from available confidence intervals, standard errors, *p* values, or other reported statistics, in accordance with Cochrane recommendations.

## 3. Results

### 3.1. Study Selection

**Figure 1:**
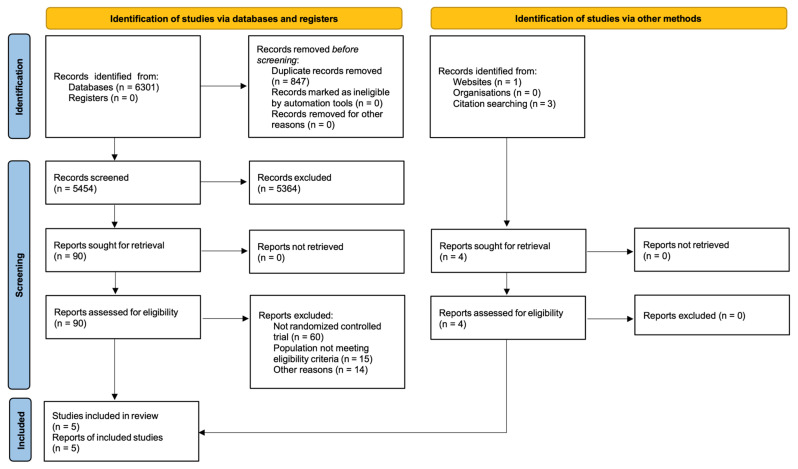
PRISMA flow diagram.

A total of 6301 records were identified through database searching, and 4 additional records were identified through citation searching. After removal of 847 duplicates, 5454 records were screened by title and abstract. Ninety full-text reports identified through database searching were assessed for eligibility, of which 89 were excluded. The main reasons for exclusion were ineligible study design (n = 60), ineligible population (n = 15), and other reasons (n = 14). Four additional reports identified through citation searching met the eligibility criteria. Ultimately, five randomized controlled trials were included in both the qualitative synthesis and the meta-analysis [29,30,31,32,33]. The study selection process is shown in Figure 1.

### 3.2. Study Characteristics

The main characteristics of the included studies are summarized in Table 1. Of the five randomized controlled trials included [29,30,31,32,33], two studies were conducted in Japan [30,31], two in German-speaking European settings [29,33], and one in Canada [32]. A total of 572 participants were randomized. Based on the studies reporting baseline demographic data, women represented 66.8% of the sample (375/561), and mean age ranged from 38.3 to 47.9 years. The study populations included adults with chronic pain or chronic pain-related conditions, including chronic low back pain, neck/shoulder pain and stiffness, frequent headaches, recurrent or heterogeneous pain presentations, and adults with arthritis or diabetes recruited for PROM-based symptom outcomes.

All studies were conducted in non-hospital or community-based contexts and recruited participants mainly through web-based, community, workplace, or outpatient channels. Outcome assessment was performed at baseline and posttreatment in all trials, with intermediate assessment points in some studies. Intervention duration ranged from 4 to 12 weeks. All interventions were delivered through smartphone-based conversational systems and were fully automated; four were rule-based and one was AI-driven. Comparators included usual care, waitlist conditions, or no-treatment controls [29,30,31,32,33].

### 3.3. Results of Individual Studies

Intervention characteristics and individual study findings are summarized in Table 2. Across all included studies [29,30,31,32,33], the interventions were fully automated and task-oriented. In terms of dialogue management, four interventions [29,30,31,33] were rule-based, whereas the intervention evaluated by MacNeill et al. [32] was AI-driven. The interventions were self-guided digital conversational programs that delivered psychoeducation, behavioral guidance, symptom monitoring, coping strategies, or brief exercise-based content. Two Japanese studies [30,31] used the LINE platform to deliver daily micro-interventions focused on exercise, posture, and symptom self-management. The two European studies [29,33] evaluated coaching-style chatbots aimed at pain self-management or frequent headache management, whereas the Canadian trial [32] assessed a modified version of the Wysa chatbot targeting mental health symptoms in adults with arthritis or diabetes.

The included studies assessed different primary outcomes. Anan et al. [30] reported significant between-group improvements in subjective pain severity in favor of the intervention. Itoh et al. [31] found no significant between-group effect for the primary outcome of work productivity, although some secondary outcomes favored the intervention, including subjective improvement in chronic low back pain and health-related quality of life. Hauser-Ulrich et al. [29] did not find significant between-group differences for the primary outcome of pain-related impairment or for pain intensity and general well-being, although the intervention group showed better working alliance bond scores. MacNeill et al. [32] reported significant between-group improvements in depression and anxiety, but not stress. Ulrich et al. [33] found significant improvements in mental well-being and in several secondary mental health outcomes, including depression, anxiety, and stress. Because outcome measures and target domains varied substantially across trials, several additional PROMs were synthesized narratively rather than quantitatively.

### 3.4. Quality Assessment and Risk of Bias

Quality assessment results are summarized in Table 1. On the Downs and Black checklist, methodological quality ranged from 14/28 to 19/28 across the included trials. Ulrich et al. [33] received the highest score (19/28), followed by Anan et al. [30] (18/28), Itoh et al. [31] (17/28), MacNeill et al. [32] (17/28), and Hauser-Ulrich et al. [29] (14/28). Reporting completeness assessed with the reduced CONSORT-EHEALTH checklist ranged from 14/21 to 20/21 items. The highest score was observed in Anan et al. [30] (20/21), followed by Hauser-Ulrich et al. [29] and Ulrich et al. [33] (19/21 each), MacNeill et al. [32] (16/21), and Itoh et al. [31] (14/21). Items related to the identification of the digital nature of the intervention, platform description, recruitment setting, comparator description, outcomes, and trial registration were generally well reported. In contrast, sample size calculation, intervention access and availability, personalization details, adherence, blinding, and ethics/privacy or safety reporting were less consistently addressed.

According to the RoB 2 assessment, three trials [29,30,31] were judged to be at high risk of bias overall and two trials [32,33] were judged to raise some concerns. The domains contributing most often to these judgments were missing outcome data (D3) and measurement of the outcome (D4), with additional concerns in deviations from intended interventions (D2) and selection of the reported result (D5) in several studies.

### 3.5. Results of Meta-Analysis

**Figure 2:**
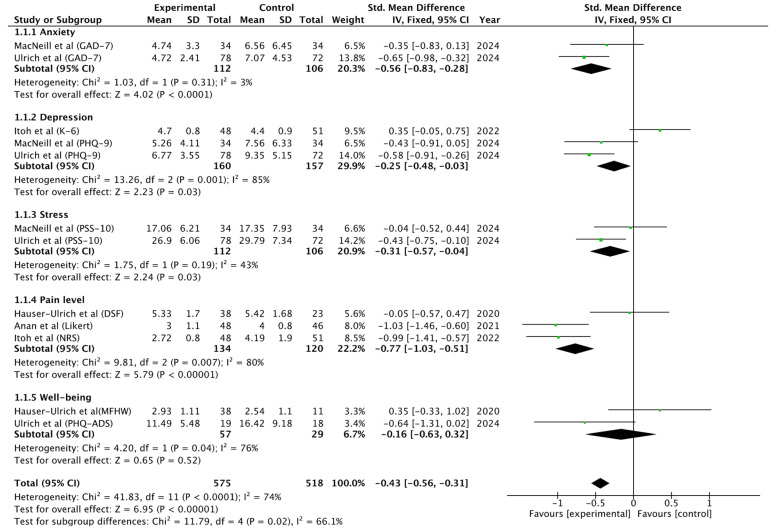
Forest plot [29,30,31,32,33].

Meta-analysis results are shown in Figure 2. A total of 12 comparisons across five PROM domains were pooled: anxiety, depression, stress, pain level, and well-being. Given the clinical heterogeneity among these outcomes, the overall pooled analysis was considered exploratory. After harmonizing outcome direction so that negative effect sizes favored the experimental intervention, the pooled estimate showed a statistically significant benefit of conversational agent interventions compared with control conditions (SMD = −0.43; 95% CI −0.55 to −0.31; I^2^ = 74%; Z = 6.91, *p* < 0.00001).

Subgroup analyses showed statistically significant effects favoring the intervention for anxiety (SMD = −0.56; 95% CI −0.83 to −0.28; I^2^ = 3%), depression (SMD = −0.25; 95% CI −0.48 to −0.03; I^2^ = 85%), stress (SMD = −0.29; 95% CI −0.56 to −0.03; I^2^ = 53%), and pain level (SMD = −0.77; 95% CI −1.03 to −0.51; I^2^ = 80%). No statistically significant pooled effect was observed for well-being (SMD = −0.16; 95% CI −0.63 to 0.32; I^2^ = 76%). Differences between outcome subgroups were statistically significant (Chi^2^ = 11.97, df = 4, *p* = 0.02; I^2^ = 66.6%), suggesting that the magnitude of effect differed across PROM domains. These findings should nevertheless be interpreted cautiously given the substantial heterogeneity observed in several subgroups and in the overall pooled analysis.

The GRADE assessment showed a consistent direction of effect favoring conversational agents (Figure S2). The certainty of evidence was rated as ‘Low’ for pain intensity (SMD −0.77) and anxiety (SMD −0.56), primarily due to serious risk of bias and clinical indirectness. For depression, stress, and well-being, certainty was rated as ‘Very Low’. These ratings reflect the high heterogeneity (I^2^ up to 85%) and imprecision typical of this emerging digital field. Despite these limitations, the findings suggest the potential for beneficial effects of conversational agents on selected outcomes; however, these results should be considered preliminary and hypothesis-generating, warranting further high-quality and standardized research.

## 4. Discussion

The primary objective of this systematic review and meta-analysis was to evaluate the effectiveness of CAs in adults with chronic pain, based on evidence from randomized controlled trials, while characterizing the intervention features and methodological variables associated with clinical outcomes. By synthesizing data from included trials involving 572 participants, our findings demonstrate that CA-based interventions provide a potencial benefit in improving various PROMs, yielding a overall pooled exploratory effect (SMD = −0.43; 95% CI −0.55 to −0.31) being in line with previous studies which analyze the use of technologic intelligence in clinical medicine [34]. However, these results must be interpreted as preliminary exploratory signals rather than definitive evidence of efficacy. While the overall trend is encouraging, the substantial heterogeneity (I^2^ = 74%) and the low certainty of evidence suggest that the observed effects should be interpreted cautiously and are likely influenced by intervention design and population context. Overall, fully automated digital dialogue systems may have potential as adjunctive tools in the multidimensional management of chronic pain, particularly in supporting patient-centered monitoring and self-management; however, these findings should be considered preliminary.

Participants were predominantly female (66.8%), with mean ages ranging from 38 to 48 years, consistent with the global epidemiology of chronic pain [3,4]. The included studies covered clinically heterogeneous conditions, including common musculoskeletal disorders such as low back and neck pain, as well as headache and pain-related symptoms in people with diabetes or arthritis. This broader scope reflects the complexity of chronic pain, which involves physical symptoms, psychosocial factors, and economic impacts [35]. It also suggests that the effects of CA-based interventions may depend on the specific pain condition, the therapeutic content delivered, the frequency of interaction, and the characteristics of the target population.

Effectiveness of the interventions appeared to be related, at least in part, to delivery format and interaction frequency. Programs ranged from 4 to 12 weeks, with the most consistent improvements in pain intensity observed in studies using frequent digital interactions [29,30,31,32,33]. In particular, rule-based systems delivered through familiar messaging platforms, such as LINE, may have provided low-burden behavioral guidance, exercise prompts, and symptom-related support in daily life. This suggests that short, structured, and repeated digital interactions could be useful for reinforcing self-management behaviors in chronic pain. These features may therefore be relevant when designing future CA-based interventions, particularly those intended to support sustained engagement in daily life [20].

In comparison with the broader literature on artificial intelligence and digital health [36], the CAs identified in this review represent a specific form of fully automated and largely standalone digital support. Although many contemporary AI-driven interventions are increasingly integrated into blended care models involving clinician support, the studies included here focused mainly on automated systems. Importantly, most interventions were rule-based rather than AI-driven, which represents a relevant conceptual distinction from a clinical and technological perspective. Rule-based systems may offer advantages in terms of standardization, reproducibility, transparency, and safety, particularly when delivering health-related guidance to individuals with chronic pain. In contrast, AI-driven systems may provide greater adaptability and personalization through more dynamic interactions. Due to the limited number of available studies, both approaches were considered together in the present review, which may have contributed to additional conceptual heterogeneity [37].

Regarding the quantitative synthesis, the overall pooled effect was consistent with previous evidence in digital health suggesting small-to-moderate effects for automated interventions [38]. The largest effect was observed for pain intensity (SMD = −0.77), while significant but smaller effects were found for anxiety, depression, and stress. These findings may suggest differential effects across outcome domains, with potentially greater impact on symptom-specific or behaviorally targeted outcomes compared with broader constructs. In contrast, no statistically significant effect was observed for general well-being (SMD = −0.16), possibly due to short intervention durations, variability in outcome measures, or the multidimensional nature of this construct. The overall pooled effect should be interpreted cautiously, as it combines clinically distinct outcomes across different domains. Therefore, this estimate is exploratory in nature and should not be interpreted as evidence of a uniform effect across all outcomes.

### Limitations

The findings of this systematic review should be interpreted with caution due to several constraints. First, the limited number of studies and modest sample sizes reduce generalizability and statistical power. Second, we observed substantial heterogeneity, likely due to diverse pain etiologies and varying intervention designs. In addition, the included studies encompassed both rule-based and AI-driven conversational agents, which may differ substantially in terms of adaptability, personalization, and underlying mechanisms of action, potentially contributing to additional conceptual heterogeneity. To address this, a random-effects model was applied to provide more conservative estimates. Third, the GRADE assessment indicated a low certainty of evidence, driven by serious risk of bias, clinical indirectness, and imprecision. Finally, the lack of long-term follow-up limits conclusions regarding the durability of effects. Overall, these findings should be considered preliminary and hypothesis-generating. Future research should prioritize larger, well-designed randomized controlled trials with standardized intervention reporting, clearer differentiation between conversational agent architectures, and longer follow-up periods to better establish the clinical value of conversational agents.

## 5. Conclusions

This systematic review and meta-analysis suggests that conversational agent-based interventions may have the potential to improve the profile of chronic-pain conditions, including patient-reported outcomes. However, these findings should be interpreted cautiously due to substantial heterogeneity and risk of bias across studies, and the low certainty of evidence identified in the GRADE assessment. Therefore, the current evidence should be considered preliminary and hypothesis-generating rather than confirmatory.

The observed effects appear broadly consistent with findings from the wider digital health literature, supporting the possible role of fully automated conversational agents as scalable, low-burden tools for symptom monitoring and self-management, particularly as adjunctive support during long waiting periods for specialist consultations.

Future research should prioritize adequately powered randomized controlled trials with standardized reporting of AI architectures, longer follow-up periods, and consistent assessment of functional and work-related outcomes. Such efforts will be necessary to clarify the long-term clinical relevance and implementation potential of conversational agents in diverse chronic pain populations.

## Figures and Tables

**Table 1:** Study characteristics.

Study (Year)	Country	Pain Population	Sample (% Female)	Age (Mean ± SD)	Risk of Bias	Quality
Hauser-Ulrich et al. (2020) [29]	Switzerland	Mixed chronic pain aetiologias	EG: 59 (86%) CG: 43 (72%)	EG: 42.97 ± 12.17 CG: 44.88 ± 13.50	High risk	14/28
Anan et al. (2021) [30]	Japan	Work associated musculoskeletal pain	EG: 48 (19%) CG: 46 (28%)	EG: 41.8 ± 8.7 CG: 42.4 ± 8.0	High risk	18/28
Itoh et al. (2022) [31]	Japan	Chronic low back pain	EG: 48 (44%) CG: 51 (45%)	EG: 47.9 ± 10.2 CG: 46.9 ± 12.3	High risk	17/28
MacNeill et al. (2024) [32]	Canada	Different chronic etiologies	EG: 41 (69%) CG: 38 (69%)	42.87 ± 11.27	Some concerns	17/28
Ulrich et al. (2024) [33]	Switzerland, Germany, Austria	Chronic headache	EG: 110 (87.3%) CG: 88 (86.4%)	EG: 39.03 ± 11.46 CG: 38.28 ± 12.82	Some concerns	19/28

CG: Control Group; EG: Experimental Group; SD: Standard Deviation.

**Table 2:** Study interventions.

Study (Year)	Communication Platform (app); Conversational Agent Type	Interaction Modalities (Input/Output)	Key Components	Comparator	Outcomes	Main Results
**Hauser-Ulrich et al. (2020) [29]**	Smartphone app (SELMA); text-based healthcare chatbot	Input: Mixed (fixed-choice + free-text) Output: Mixed (written + multimedia)	CBT-based pain self-management, psychoeducation, coping strategies	Waitlist	Pain-related impairment (BPI) Pain intensity (DSF), general well-being (MFHW), working alliance (WAI-SR bond)	Working alliance EG > CG (*p* = 0.005) NS results: Pain-related impairment 71% intervention adherence Usefulness: 5.47/7 Usability 6.34/7
**Anan et al. (2021) [30]**	Mobile messaging app (LINE); conversational exercise-support chatbot	Input: Fixed-choice Output: Mixed (written + visual)	Exercise support, stretching, posture, mindfulness	Usual care: regular workplace exercise routine	Pain severity of neck/shoulder stiffness/pain or low back pain (1–5 scale), Presence of severe pain according to subjective pain scores (score 4–5), Achievement of subjective symptom improvement (improved/slightly improved)	Pain severity EG < CG (*p* < 0.001) 92% intervention adherence NR Acceptability
**Itoh et al. (2022) [31]**	Mobile messaging app (LINE); conversational exercise-therapy chatbot	Input: NR Output: Mixed (written + multimedia)	Patient education, exercise therapy, posture/core alignment	Usual medical care: routine CLBP medical care	Work productivity (QQ method) Severe pain at 12 weeks (score 4–5); subjective improvement (improved/slightly improved) at 12 weeks Work productivity (WPAI-GH), low back pain and shoulder stiffness (NRS), subjective CLBP improvement (1–5 scale), disease-specific QoL (RDQ-24), health-related QoL (EQ-5D-5L), kinesiophobia (TSK-11), depression/psychological distress (K-6)	Subjective CLBP improvement EG < CG (*p* = 0.04) Health-related QoL EG > CG (*p* = 0.03) NS results: Work productivity, Low back pain, Disease-specific QoL, Depression/psychological distress 65–77% intervention adherence NR Acceptability
**MacNeill et al. (2024) [32]**	Smartphone app (Wysa); mental health chatbot	Input: Mixed (free-text + fixed-choice) Output: Mixed (written + multimedia)	Mental health support, self-care exercises, check-ins/reminders	No-treatment control	Depression (PHQ-9), anxiety (GAD-7), stress (PSS-10)	Depression EG < CG (*p* < 0.001) Anxiety EG < CG (*p* < 0.001) NS results: Stress NR Adherence “Generally positive; easy to use, convenient and accessible. Some conversational issues reported”
**Ulrich et al. (2024) [33]**	Smartphone coaching app (BalanceUP); conversational agent coach	Input: Mixed (fixed-choice + free-text) Output: Mixed (written + multimedia)	Headache coaching, psychoeducation, behavior change/action planning, relaxation	Waitlist	Mental well-being (PHQ-ADS), depression (PHQ-9), anxiety (GAD-7), somatic symptoms (PHQ-15), stress (PSS-10), headache management self-efficacy (HMSE-G-SF), BCTs application (HAPA), absenteeism/presenteeism (MIDAS), cognitive & behavioural pain coping (FESV)	Mental well-being EG < CG (*p* < 0.001) Depression EG < CG (*p* < 0.001) Anxiety EG < CG (*p* = 0.007) Stress EG < CG (*p* = 0.003) 64.8% intervention adherence Usefulness 4.00/5 Usability 3.56/5

BCTs: Behavior Change Techniques; BPI: Brief Pain Inventory; CBT: Cognitive Behavioral Therapy; CG: Control group; CLBP: Chronic Low Back Pain; DSF: Deutscher Schmerzfragebogen (German Pain Survey); EG: Experimental group; EQ-5D-5L: EuroQoL 5 Dimensions 5 Level; FESV: Fragebogen zur Erfassung der Schmerzverarbeitung; GAD-7: General Anxiety Disorder Scale-7; HAPA: Health Action Process Approach; HMSE-G-SF: German short form of the Headache Management Self-Efficacy Scale; K-6: Kessler Screening Scale for Psychological Distress; MFHW: Marburger Screening for Habitual Well-being; MIDAS: Migraine Disability Assessment; NR: Not reported; NRS: Numerical Rating Scale; NS: Not significant; PHQ-15: Patient Health Questionnaire-15; PHQ-9: Patient Health Questionnaire-9; PHQ-ADS: Patient Health Questionnaire Anxiety and Depression Scale; PSS-10: Perceived Stress Scale-10; QoL: Quality of Life; QQ method: Quantity and Quality method; RDQ-24: Roland-Morris Disability Questionnaire; TSK-11: Tampa Scale for Kinesiophobia; WAI-SR: Working Alliance Inventory–Short Revised; WPAI-GH: Work Productivity and Activity Impairment Questionnaire-General Health.

## Data Availability

No new data were created or analyzed in this study. Data sharing is not applicable to this article.

## Supplementary Materials

The following supporting information can be downloaded at: https://www.mdpi.com/article/10.3390/healthcare14101360/s1, Figure S1. Search strategy; Figure S2. GRADE evidence quality ratings.

## Abbreviations

The following abbreviations are used in this manuscript:

PROMs	Patient-reported outcome measures
CAs	Conversational agents
RoB 2	Cochrane Risk of Bias 2 tool
RevMan	Review Manager
SMDs	Standardized mean differences
AI	Artificial Intelligence
