# Supplementary material for: Effectiveness of Conversational Agents on Patient-Reported Outcomes in Chronic Pain Management: A Systematic Review and Meta-Analysis
Source: Healthcare (Basel). 2026 May 15;14(10):1360. doi: 10.3390/healthcare14101360 (PMC13206324; doi:10.3390/healthcare14101360)
Supplement: Supplementary file 1 [file healthcare-14-01360-s001.zip › healthcare-4311844-supplementary.pdf]

Figure S1. Search strategy.

Database	Search Query	
**PubMed**	#1	((Chatbot OR chatterbot OR chatter robot OR artificial intelligence OR “conversational AI” OR “conversational agency” OR “virtual agent” OR “conversational agents” OR “bot” OR “Speech recognition software” OR “Conversational adj1 agent” OR “embodied adj2 agent” OR chatbot* OR avatar* OR “dialog* adj1 system” OR “voice recognition software” OR (virtual adj1 (assistan* OR nurs* OR patient OR coach* OR agent)) OR (relation* adj1 agent) OR assistance technol* OR (intelligent adj2 assistan*) OR (digital adj2 assistan*) OR “natural language interface” OR “interactive computer agent” OR “computer-assisted instruction” OR “natural language communication” OR “natural language understanding” OR “unconstrained natural language processing”)
	#2	(“chronic pain” OR “noncancer pain” OR “musculoskeletal pain” OR osteoarthritis OR arthritis OR rheumatoid OR “myalgia” OR “musculoskeletal pains” OR “muscle pain” OR “myofascial pain syndrome” OR “trigger point”))
	#1 AND #2	
**Web of Science**	#1 AND #2<br>AND<br>("Clinical trial" OR RCT OR “RCTs” OR "Randomized Control Trial" OR "Randomised Control Trial" OR "Randomised Controlled Trial" OR “randomized controlled trial“ OR “controlled clinical trial”)	
**Scopus**	#1 AND #2<br>AND<br>("Clinical trial" OR RCT OR “RCTs” OR "Randomized Control Trial" OR "Randomised Control Trial" OR "Randomised Controlled Trial" OR “randomized controlled trial“ OR “controlled clinical trial”)	

Figure S2. GRADE evidence quality ratings.

Certainty assessment							Summary of results				
							Study event rates (%)		Relative effect (95% CI)	Anticipated absolute effects	
Participants (studies) follow-up	Risk of bias	Inconsistency	Indirectness	Imprecision	Publication bias	Overall certainty of evidence	With interventions without automated conversational agents or no intervention	With fully automated conversational agents		Risk with interventions without automated conversational agents or no intervention	Risk difference with fully automated conversational agents
**PROMs - Anxiety**											
218 (2 randomized controlled trials (RCTs))	Serious[a]	Not serious	Serious[b]	Not serious	None	⊕⊕◯◯ Low[a,b]	106	112	-	-	SMD **0.56 lower** (0.83 lower to 0.28 lower)
**PROMs - Depression**											
317 (3 randomized controlled trials (RCTs))	Serious[c]	Very serious[d]	Serious[e]	Serious[f]	None	⊕◯◯◯ Very low[c,d,e,f]	157	160	-	-	SMD **0.25 lower** (0.48 lower to 0.03 lower)
**PROMs - Stress**											
218 (2 randomized controlled trials (RCTs))	Serious[g]	Not serious	Serious[h]	Serious[i]	None	⊕◯◯◯ Very low[g,h,i]	106	112	-	-	SMD **0.31 lower** (0.57 lower to 0.04 lower)
**PROMs - Pain level**											
254 (3 randomized controlled trials (RCTs))	Serious[j]	Serious[k]	Not serious	Not serious	None	⊕⊕◯◯ Low[j,k]	120	134	-	-	SMD **0.77 lower** (1.03 lower to 0.51 lower)

Certainty assessment							Summary of results				
**PROMs - Well-being**											
86 (2 randomized controlled trials (RCTs))	Serious[l]	Serious[m]	Not serious	Serious[n]	None	⊕◯◯◯ Very low[l,m,n]	29	57	-	-	SMD **0.16 lower** (0.63 lower to 0.32 higher)

CI: Confidence interval; **SMD:** standardized mean difference

Explanations

a. Downgraded one level for serious risk of bias because the contributing trials were not at low risk of bias overall. Important concerns included lack of blinding/open-label design and missing outcome data, with additional concerns related to outcome measurement, which reduce confidence in the anxiety estimate.

b. Downgraded one level for serious indirectness because the contributing studies differed importantly in population and intervention context. One trial included adults with frequent headaches, whereas the other included people with chronic diseases (arthritis or diabetes), and the conversational interventions and control conditions were not fully comparable. Therefore, the pooled anxiety estimate may not directly represent a single, well-defined chronic pain population or a uniform intervention model.

c. Downgraded one level for serious risk of bias because the contributing studies had important methodological limitations and were not at low risk of bias overall. Key concerns included missing outcome data and outcome measurement, with additional concerns related to deviations from intended interventions and selection of the reported result, which reduce confidence in the depression estimate.

d. Downgraded two levels for very serious inconsistency because heterogeneity was very high ($I^2 = 85\%$), and the study effects differed importantly in direction and magnitude. One trial showed an effect in the opposite direction or close to no effect, whereas the others favored the intervention. Differences in populations, intervention formats, comparator conditions, and outcome measurement are likely to have contributed to this substantial between-study variability.

e. Downgraded one level for serious indirectness because this outcome was derived from studies including clinically diverse populations and different intervention models, and one study assessed psychological distress rather than depression specifically. Therefore, the evidence does not fully correspond to a single, directly comparable depression construct across all included trials.

f. Downgraded one level for serious imprecision because only a small number of studies contributed to this outcome, and the confidence interval was close to the line of no effect (-0.48 to -0.03). This means that the true effect could range from a small-to-moderate benefit to a trivial effect, which limits confidence in the magnitude and stability of the estimate.

g. Downgraded one level for serious risk of bias because the contributing studies had important methodological limitations and were not at low risk of bias overall. The main concerns were missing outcome data and measurement of the outcome, with additional concerns related to deviations from intended interventions and selection of the reported result.

h. Downgraded one level for serious indirectness because the contributing studies differed importantly in population and intervention context. One trial included adults with frequent headaches, whereas the other included people with chronic diseases such as arthritis or diabetes, and the conversational interventions and comparator conditions were not fully comparable. Therefore, the estimate does not directly represent a single, clearly defined chronic pain population or a uniform intervention model.

i. Downgraded one level for serious imprecision because only a small number of studies contributed to this outcome, and the confidence interval was close to the line of no effect (-0.56 to -0.03). This means that the true effect could range from a moderate benefit to a trivial effect, which limits confidence in the magnitude and stability of the estimate.

j. Downgraded one level for serious risk of bias because the contributing studies were not at low risk of bias overall. The main concerns were missing outcome data and measurement of the outcome, with additional concerns related to deviations from intended interventions and selection of the reported result, which reduce confidence in the pain estimate.

k. Downgraded one level for serious inconsistency because substantial statistical heterogeneity was observed ($I^2 = 80\%$). This variability is likely related to differences in pain populations, intervention designs, comparator conditions, and outcome measures across studies, which reduce confidence in the consistency of the pooled pain estimate.

l. Downgraded one level for serious risk of bias because the contributing studies were not at low risk of bias overall. The main concerns were missing outcome data and measurement of the outcome, with additional concerns related to deviations from intended interventions and selection of the reported result, which reduce confidence in the well-being estimate.

m. Downgraded one level for serious inconsistency because substantial heterogeneity was observed ($I^2 = 76\%$). Differences in populations, intervention characteristics, comparator conditions, and the way well-being was conceptualized and measured likely contributed to this variability, reducing confidence in the consistency of the pooled estimate.

n. Downgraded one level for serious imprecision because the confidence interval crossed the line of no effect (-0.63 to 0.32), and only a small number of studies contributed to this outcome. This limits confidence in whether the intervention provides a true benefit for well-being and in the precision of the effect estimate.
